# Histone demethylase LSD1 regulates bone mass by controlling WNT7B and BMP2 signaling in osteoblasts

**DOI:** 10.1038/s41413-018-0015-x

**Published:** 2018-04-26

**Authors:** Jun Sun, Joerg Ermann, Ningning Niu, Guang Yan, Yang Yang, Yujiang Shi, Weiguo Zou

**Affiliations:** 1 State Key Laboratory of Cell Biology, CAS Center for Excellence in Molecular Cell Science, Shanghai Institute of Biochemistry and Cell Biology, Chinese Academy of Sciences, University of Chinese Academy of Sciences, 320 Yueyang Road, Shanghai, 200031 China; 2 Division of Rheumatology, Immunology, and Allergy, Brigham and Women’s Hospital, Harvard Medical School, Boston, MA 02115 USA; 3 Newborn Medicine Division, Boston Children’s Hospital and Department of Cell Biology, Harvard Medical School, Boston, MA 02115 USA

## Abstract

Multiple regulatory mechanisms control osteoblast differentiation and function to ensure unperturbed skeletal formation and remodeling. In this study we identify histone lysine-specific demethylase 1(LSD1/KDM1A) as a key epigenetic regulator of osteoblast differentiation. Knockdown of LSD1 promoted osteoblast differentiation of human mesenchymal stem cells (hMSCs) in vitro and mice lacking LSD1 in mesenchymal cells displayed increased bone mass secondary to accelerated osteoblast differentiation. Mechanistic in vitro studies revealed that LSD1 epigenetically regulates the expression of WNT7B and BMP2. LSD1 deficiency resulted in increased BMP2 and WNT7B expression in osteoblasts and enhanced bone formation, while downregulation of WNT7B- and BMP2-related signaling using genetic mouse model or small-molecule inhibitors attenuated bone phenotype in vivo. Furthermore, the LSD1 inhibitor tranylcypromine (TCP) could increase bone mass in mice. These data identify LSD1 as a novel regulator of osteoblast activity and suggest LSD1 inhibition as a potential therapeutic target for treatment of osteoporosis.

## Epigenetics: Taking the brakes off bone formation

A molecular brake controlling the differentiation of bone-forming osteoblasts has been identified, which could provide a new therapeutic target for osteoporosis. LSD1 regulates the expression of various genes by interfering with the ability of the cellular machinery to bind to DNA and transcribe it. A previous study had suggested that inhibiting LSD1 in stem cells from human fat promoted the formation of new osteoblasts. To further investigate its role in bone development, Weiguo Zou at the Shanghai Institute of Biochemistry and Cell Biology and colleagues engineered mice which lacked LSD1 in their mesenchymal cells–precursors of bone, muscle and fat cells—the animals had increased numbers of osteoblasts and greater bone mass. Further experiments revealed that blocking LSD1 resulted in increased expression of two osteoblast-stimulating factors, although it may also influence bone resorbing osteoclasts.

## Introduction

Bone is a dynamic organ that supports locomotive activity, maintains blood calcium levels, serves as a reservoir for hematopoietic stem cells, and houses the brain and spinal cord. The maintenance of bone is accomplished by continuous remodeling throughout life via the balanced activity of mesenchymally derived osteoblasts and hematopoietically derived osteoclasts.^1^ Osteoblasts are the bone-forming cells, which synthesize collagens and proteins such as osteocalcin and osteopontin to form bone matrix while osteoclasts resorb bone in response to microfractures. The differentiation of osteoblasts from mesenchymal progenitors is regulated by multiple developmental signals, transcription factors (such as Runx2 and Osterix) and cytokines.^2^

LSD1 is a flavin-containing amino oxidase that specifically catalyzes the demethylation of monomethylated and dimethylated histone 3 lysine 4 (H3K4) residues and generally functions as a transcriptional repressor.^3^ LSD1 has also been shown to promote nuclear hormone receptor induced transcription via H3K9me1/me2 demethylation.^4,5^ Germline deletion of *Lsd1* in mice results in embryonic lethality and embryonic stem cells lacking LSD1 show impaired differentiation potential indicating an important role for LSD1 during embryogenesis.^6,7^ LSD1 also orchestrates the emergence and differentiation of hematopoietic stem cells, ^8–10^ regulates fat metabolism and modulates p53 signaling pathway.^11,12^ In addition, LSD1 has been associated with multiple human malignancies including prostate cancer, bladder cancer, leukemia, and others.^13,14^ Because of the important role of LSD1 in these diseases, pharmacological LSD1 inhibitors have been developed.^15–17^ A recent study showed that inhibition of LSD1 in human adipose-derived stem cells (hASCs) using LSD1 inhibitors enhanced osteoblastogenesis,^18^ but the precise in vivo role of LSD1 in bone development and remodeling remains to be determined.

Here we explored the function of LSD1 in osteoblasts using a genetic mouse model and mechanistic in vitro studies. Mice with conditional deletion of *Lsd1* in mesenchymal cells exhibited an enhanced bone mass phenotype with increased osteoblast numbers. LSD1 negatively regulated the expression of BMP2 and WNT7B via demethylation resulting in increased BMP2-induced BMP signaling and WNT7B induced mTOR signaling in LSD1-deficient osteoblasts. Moreover, inhibition of LSD1 using the small inhibitor tranylcypromine (TCP) increased bone mass in mice. Together, these findings provide strong in vivo evidence for the role of LSD1 as a repressor of osteoblastogenesis through repressing BMP2 and WNT7B expression and reveal it as a potential therapeutic target for osteoporosis.

## Results

### LSD1 inhibits osteoblast differentiation of hMSCs in vitro

**Fig. 1:**
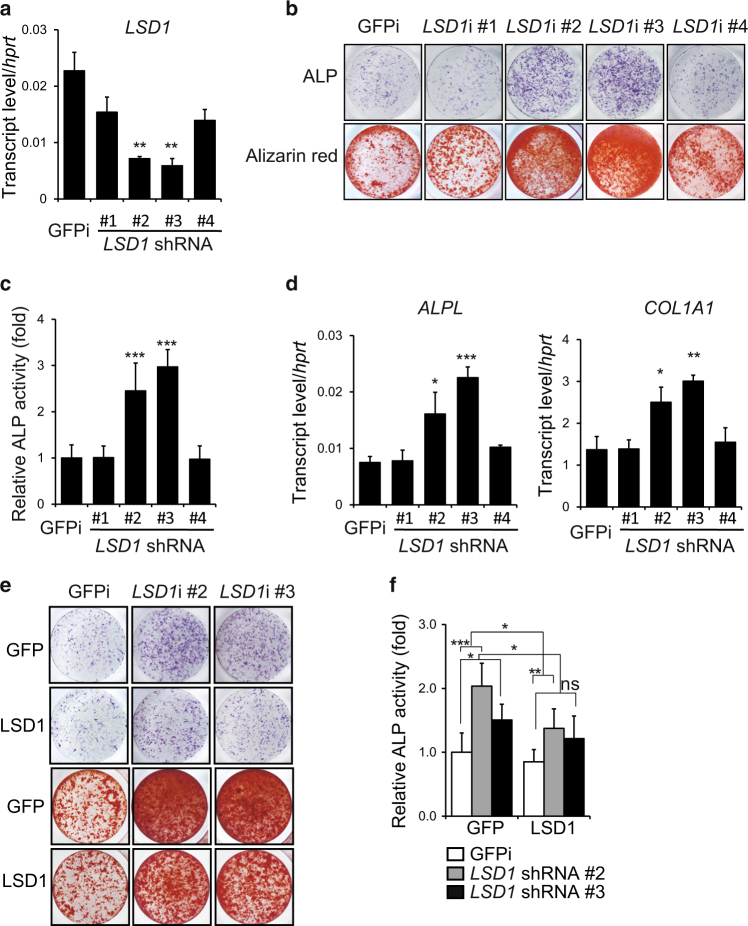
LSD1 inhibits osteoblastogenesis of hMSCs in vitro. **a** RT-PCR analysis of the knockdown efficiency of *LSD1* at day7 during hMSCs differentiation, *n* = 3, unpaired *t*-test, **P < 0.01. **b**, **c** ALP staining (**b**, upper panel), alizarin red staining (**b**, lower panel), and ALP quantification (**c**) of hMSCs infected with *GFP*- or *LSD1*-specific shRNA lentivirus at day7 or day14. Data are presented as mean ± SD (*n* = 6), unpaired *t*-test, **P < 0.01, ***P < 0.001. **d** RT-PCR analysis of *ALPL* and *COLLA1* expression at day7 during hMSCs differentiation. *n* = 3, unpaired *t*-test, *P < 0.05, **P < 0.01, ***P < 0.001. **e**, **f** Osteoblast differentiation was analyzed by ALP staining (**e**, upper panel), alizarin red staining (**e**, lower panel), and ALP quantification (**f**) after infection of hMSCs with indicated shRNA and *LSD1* expression lentiviruses and subsequent culture for 7 days or 14 days. Data in **f** are presented as mean ± s.d (*n* = 6). ANOVA followed by Tukey’s post hoc test was performed.*P < 0.05, **P < 0.01, ***P < 0.001, ns not significant.

We previously performed an RNAi-based loss-of-function screen in bone marrow-derived human mesenchymal stem cells (hMSCs) to identify novel regulators of osteoblast differentiation^19^ and found that knockdown of LSD1 enhanced osteoblast differentiation. To verify this result, hMSCs transduced with four different shRNA lentivirus constructs targeting human *LSD1* were cultured in osteoblast differentiation media. Knockdown of *LSD1* enhanced osteoblast differentiation, as demonstrated by increased induction of alkaline phosphatase (ALP) activity, an early marker of osteogenesis (Fig. 1a–c). Consistent with enhanced osteoblast differentiation, the expression of characteristic osteogenic marker genes including *ALP* and collagen1 alpha 1 (*COL1A1*) was increased in *LSD1* knockdown cells (Fig. 1d). Alizarin red staining confirmed increased mineralization in *LSD1* knockdown cultures at a later time point (Fig. 1b). Furthermore, overexpression of mouse *Lsd1*, which is not targeted by the human *LSD1*-specific shRNAs, abrogated the effects of *LSD1* knockdown on ALP activity and mineralization capacity (Fig. 1e, f), thereby proving the specificity of the knockdown results and confirming that knockdown of *LSD1* promotes osteoblast differentiation of hMSCs in vitro. Taken together, these data suggest that LSD1 plays a negative role in osteoblast differentiation.

### Deletion of *Lsd1* in mesenchymal progenitor cells leads to increased bone mass in mice

**Fig. 2:**
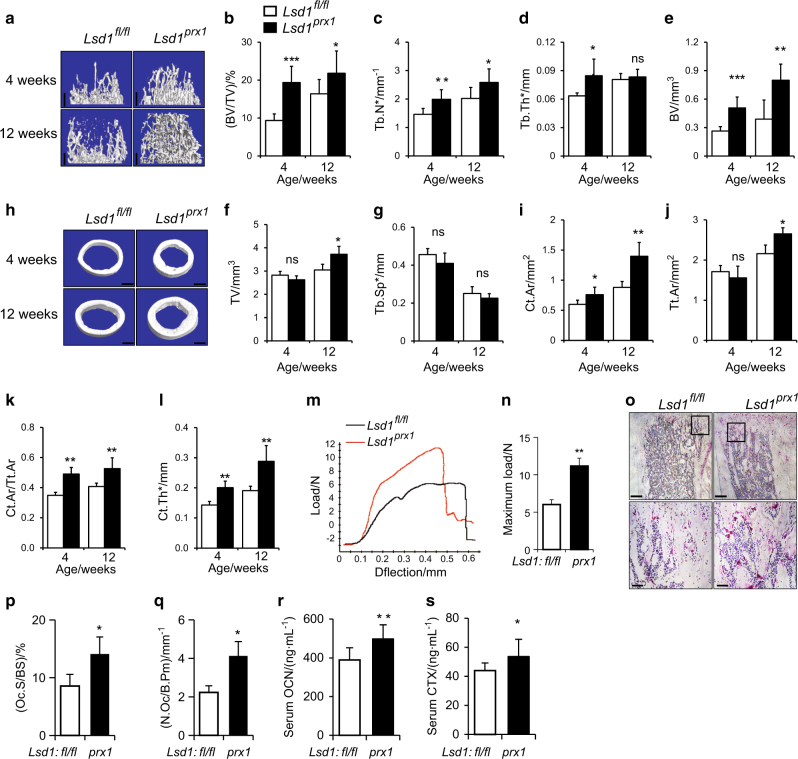
Deletion of *Lsd1* in osteoblast progenitor cells leads to increased bone mass. **a**–**l** *Lsd1*^*prx1*^ mice exhibited increased bone volume. Femurs from 4 weeks and 12 weeks female *Lsd1*^*prx1*^ and *Lsd1*^*fl/fl*^ mice were analyzed by μCT. 3D reconstructions of the trabecular bone (**a**) and midshaft cortical bone (**h**), quantitative parameters of trabecular bone (**b**–**g**) and cortical bone (**i**–**l**) were analyzed, bone volume (BV), total volume (TV), trabecular number per cubic millimeter (Tb.N), trabecular thickness (Tb.Th), trabecular separation (Tb.Sp), cortical thickness (C.Th), Tt.Ar (total cross-sectional area), and Ct.Ar (cortical bone area), *n* = 6, unpaired *t*-test, *P < 0.05, **P < 0.01, ***P < 0.001. **m**, **n** Three-point bend test was performed on femurs of 4-week-old *Lsd1*^*prx1*^ and *Lsd1*^*fl/fl*^ littermates. Representative image of load-deflection diagram demonstrating the differences in the mechanical properties of bone from control and *Lsd1*^*prx1*^ mice (**m**) and the biomechanical properties maximum load was measured during the test (**n**), *n* = 5, unpaired *t*-test, **P < 0.01. **o** Histological analysis of TRAP-positive osteoclast populations in the tibia of 4-week-old *Lsd1*^*prx1*^ and *Lsd1*^*fl/fl*^ mice. Data are representative of three independent experiments. **p**, **q** Histomorphometric analysis of bone resorption, osteoclast surface (Oc.S/BS), and osteoclast number (N.Oc/B.Pm) was shown. Statistical analysis, unpaired *t*-test, *P < 0.05. **r**, **s** The serum bone turnover marker CTX (bone resorption) and Osteocalcin (bone formation) were measured in 5-week-old *Lsd1*^*prx1*^ and *Lsd1*^*fl/fl*^ mice. *n* = 10, unpaired *t*-test, *P < 0.05, **P < 0.01. Scale bar in **a**, **h**, **o** (upper panel): 0.5 mm, **o** (lower panel): 0.1 mm.

To investigate the role of LSD1 in skeletal remodeling in vivo, we crossed mice with a floxed *Lsd1* gene (hereafter called *Lsd1*^*fl/fl*^ mice) with *Prx1*-cre mice that shows no basal phenotype^20^ to specifically delete the *Lsd1* gene in mesenchymal progenitors (*Prx1-Cre, Lsd1*^*fl/fl*^*;*, hereafter *Lsd1*^*prx1*^). LSD1 protein was largely abrogated in long bone tissue of *Lsd1*^*prx1*^ mice (Figure S1 A). *Lsd1*^*prx1*^ mice displayed approximately 20% shorter stature and 30% lower body weight compared with *Lsd1*^*fl/fl*^ littermate controls at 4 weeks of age, both genders showed the same phenotype (Figure S1 B-D). The fat store showed no significant difference between *Lsd1*^*prx1*^ mice and control mice (Figure S1 E-G). *Lsd1*^*prx1*^ mice also exhibited delayed chondrocyte/cartilage development and endochondral bone formation (Figure S1 H), indicating that LSD1 may play important roles in chondrocyte/cartilage development. We then used Microcomputed tomography (microCT) analysis to examine the bone development. *Lsd1*^*prx1*^ mice exhibited increased trabecular bone volume at 4 and 12 weeks of age (Fig. 2a–g). The trabecular number and trabecular thickness were increased in *Lsd1*^*prx1*^ mice (Fig. 2c, d). In addition to the trabecular phenotype, *Lsd1*^*prx1*^ mice also displayed increased cortical bone thickness (Fig. 2h–l). In contrast, *Prx1*-cre, *Lsd1*^*fl/+*^ mice did not show obvious bone phenotype (Figure S2 A-L). The parental bone of *Lsd1*^*prx1*^ mice also showed increased BV/TV and BMD, indicating that the intramembranous bone formation was also enhanced in *Lsd1*^*prx1*^ mice (Figure S2 M-O). We next analyzed the structural integrity of femurs from 4-week-old *Lsd1*^*prx1*^ and control mice by three-point bending tests. Consistent with the increase in bone mass, *Lsd1*^*prx1*^ femurs showed increased bone stiffness compared with *Lsd1*^*fl/fl*^ littermate controls (Fig. 2m, n). H&E staining of sections from the proximal tibias from 4-week-old mice and histomorphometry analysis confirmed increased bone mass in *Lsd1*^*prx1*^ mice (Figure S2 P-T). In addition, we observed higher osteoclast activity in *Lsd1*^*prx1*^ mice by TRAP staining of sections from the tibia (Fig. 2o–q). The serum bone turnover markers, CTX and Osteocalcin, were also increased in *Lsd1*^*prx1*^ mice (Fig. 2r, s). Taken together, these data suggest that deletion of *Lsd1* in mesenchymal cells increases bone turnover.

### Increased osteoblast activity in *Lsd1*^*prx1*^ mice

**Fig. 3:**
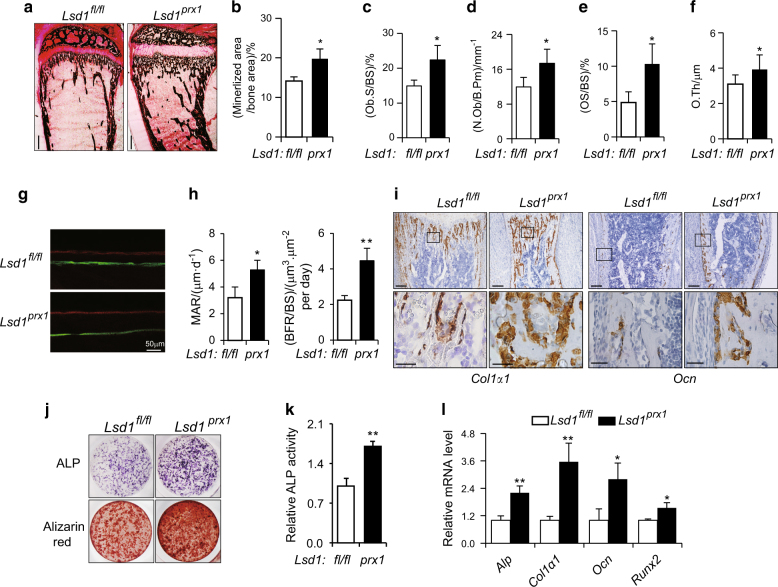
Increased osteoblast activity in *Lsd1*^*prx1*^ mice. **a** Von Kossa staining of proximal tibia from 4-week-old *Lsd1*^*prx1*^ and control mice, bar: 0.5 mm, *n* = 3. **b** Quantification of Von Kossa staining was represented as percentage of mineralized bone area determined by Image J program. **c**–**f** Bone histomorphometric analysis of proximal tibia from 4-week-old mice. The osteoid surface (OS/BS), osteoblast number (N.Ob/B.Pm), osteoblast surface (Ob./BS), and osteoid thickness (O.Th) are shown. Data represent the mean ± standard deviation (SD) of four mice, *P < 0.05. **g** Double labeling of mineralizing front with calcein and alizarin red S of tibia bone from *Lsd1*^*fl/fl*^ and *Lsd1*^*prx1*^ mice was visualized by fluorescent microscopy, *n* = 3. **h** Analysis of mineral apposition rate (MAR) and bone formation rate (BFR) in *Lsd1*^*fl/fl*^ and *Lsd1*^*prx1*^ mice. Results are presented as mean ± SD (*n* = 3), Statistical analysis, unpaired *t*-test, *P < 0.05, **P < 0.01. **i** In situ hybridization for *collagen I* (*Col I*) and *osteocalcin*(*Ocn*) in the tibia of 1-week-old *Lsd1* ^*prx1*^ and *Lsd1*^*fl/fl*^ mice. Results are representative of two independent experiments. Scale bar, upper panel: 200 μm, lower panel: 50 μm. **j** ALP and alizarin red staining in osteogenic cultures of BMSCs. For ALP staining, cells were cultured for 7 days. For alizarin red staining, cells were cultured for 14 days. **k** Quantification of ALP activity, *n* = 5, unpaired *t*-test, **P < 0.01. **l** RT-PCR analysis of osteogenesis gene expression in *Lsd1* ^*prx1*^ and *Lsd1*^*fl/fl*^ BMSC at cell culture day 7. Data are presented as mean ± s.d (*n* = 3), Statistical analysis, unpaired *t*-test, *P < 0.05, **P < 0.01.

To formally assess the bone mass phenotype in *Lsd1*^*prx1*^ mice, Von Kossa staining and histomorphometry were performed. Consistent with micro CT data, increased mineralized bone was observed in 4-week-old *Lsd1* ^*prx1*^ mice tibias (Fig. 3a, b). The bone formation parameters, including OS/BS, N.Ob/B.Pm, Ob.S/BS, and O.Th were increased in *Lsd1* ^*prx1*^ mice tibias (Fig. 3c–f). To gain further insight into whether the increased bone mass in *Lsd1*^*prx1*^ mice was due to increased bone formation, dynamic histomorphometry of tibias from 4-week-old *Lsd1*^*prx1*^ and control mice was performed. Fluorescent double labeling of the mineralizing front with calcein and alizarin red S showed an increase in mineral apposition rate (MAR) and bone formation rate (BFR) of *Lsd1*^*prx1*^ mice compared with control mice (Fig. 3g, h). Consistent with enhanced osteoblast activity, expression of the characteristic osteoblast genes osteocalcin (*Ocn*) and collagen I alpha 1 (*Col1a1*) was upregulated in the tibias of *Lsd1*^*prx1*^ mice compared with *Lsd1*^*fl/fl*^ littermate controls (Fig. 3i). To explore whether knockout of *Lsd1* promotes murine osteoblast differentiation, we performed ex vivo cultures of primary bone marrow stroma cells from *Lsd1*^*fl/fl*^ and *Lsd1*^*prx1*^ mice. Osteoblast differentiation was enhanced in *Lsd1* knockout cells as demonstrated by increased ALP activity and bone matrix formation (Fig. 3j, k), as well as upregulated expression of osteogenesis marker genes (Fig. 3l). These results indicate that the increased osteoblast differentiation and activity is responsible for the enhanced bone mass in *Lsd1*^*prx1*^ mice.

### *Wnt7b* and *Bmp2* expression is increased in *Lsd1* deleted cells

**Fig. 4:**
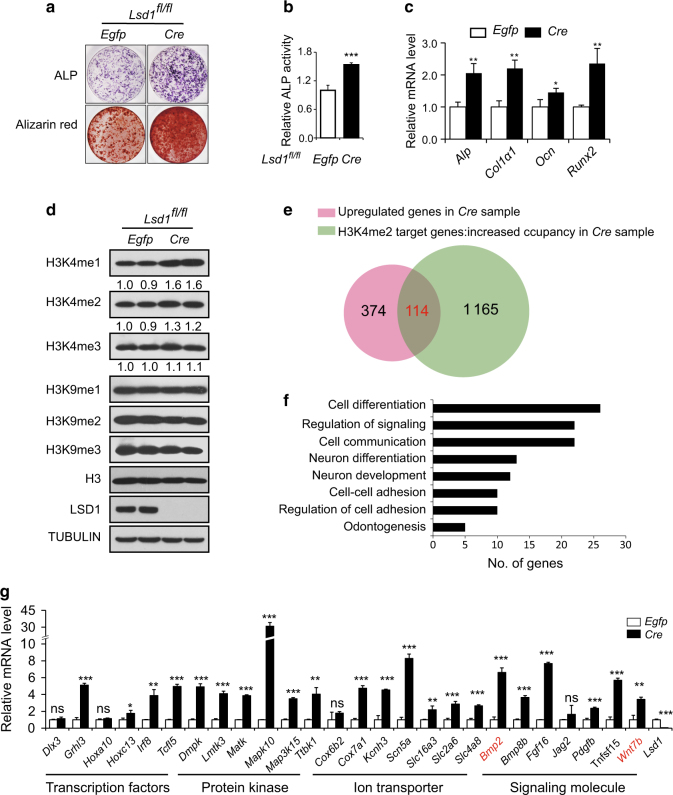
*Wnt7b* and *Bmp2* expression is increased in *Lsd1* deleted osteoblasts. **a**–**c** *Lsd1*^*fl/fl*^ calvarial cells derived from P3 mice were infected with *Cre*-lentivirus or *Egfp*-lentivirus followed by culturing in osteoblast differentiation media for 14 days. ALP staining (day 7), alizarin red staining (day 14) (**a**) and quantification of ALP activity (**b**) of calvarial cells in osteogenic cultures. Data in **b** are the mean ± SD (*n* = 6), ***P < 0.001. Unpaired *t*-test was performed. **c** mRNA expression of osteoblast marker genes at day7 was analyzed by RT-PCR, *n* = 3, unpaired *t*-test, *P < 0.05, **P < 0.01. **d** Histone H3 lysine4, lysine9 methylation, and LSD1 levels were measured by western blot. **e** Venn diagram shows the overlap of genes of increased expression level and increased H3K4me2 occupancy at the TSS region in LSD1 mutant osteoblasts. **f** Gene Ontology (GO) analysis within the 114 genes using the database for annotation, visualization, and integrated discovery (DAVID) bioinformatics tools. The enriched GO biological processes were identified and listed. The enrichment *P*-value is <0.01. **g** RT-PCR analysis of the expression levels of candidate genes in RNA-seq samples. Data in **g** represent the mean ± SD (*n* = 3) Unpaired *t*-test was performed. *P < 0.05, **P < 0.01, ***P < 0.001.

To identify genes regulated by LSD1 in osteoblasts, we infected calvarial osteoblast precursors from newborn *Lsd1*^*fl/fl*^ mice with *Cre*-virus or control *Egfp*-virus followed by culture in osteoblast differentiation medium. As expected, ALP staining on day7 and alizarin red staining on day14 were increased in *Cre*-virus infected *Lsd1*^*fl/fl*^ cells compared with *Egfp*-virus infected cells, consistent with enhanced osteoblast differentiation (Fig. 4a, b). The expression of osteoblast marker genes was also upregulated upon *Lsd1* deletion (Fig. 4c). LSD1 protein was completely deleted in *Cre*-virus infected osteoblasts and total H3K4me1/2 levels were increased compared with *Egfp*-virus transduced cells while total H3K9me1/2 levels which are also targeted by LSD1 were unchanged (Fig. 4d). Next, we performed ChIP-seq using an antibody specific for H3K4me2 as well as RNA-seq on *Lsd1*^*fl/fl*^ calvarial cells infected with *Cre or Egfp* virus. We found 1 165 genes with upregulated expression and 374 genes with increased enrichment of H3K4me2 marks in promoter regions in *Lsd1* deleted samples compared with controls (Fig. 4e). As LSD1 is an H3K4me1/2 demethylase that generally acts as a transcriptional repressor, we focused on a smaller subset of 114 genes which showed both increased mRNA expression and increased enrichment of H3K4me2 marks (Fig. 4e and table S1). Gene Ontology (GO) analysis using the database for annotation, visualization and integrated discovery (DAVID) bioinformatics tools revealed that many of these genes are involved in cell differentiation processes (Fig. 4f). Functionally, this group includes transcription factors, protein kinases, ion transporters, and signaling molecules (Fig. 4g). Differential expression of most of these genes was validated by RT-PCR (Fig. 4g). Among these genes, two secreted proteins, WNT7B and BMP2, have strong ability to induce osteoblast differentiation^21,22^ and knockdown of *Lsd1* in embryonic stem cells has been shown to upregulate *Bmp2*.^23^

Another Bmp gene, *Bmp8b*, is also upregulated in *Lsd1* knockout osteoblast. To investigate whether Bmp8b can also stimulate osteoblastogenesis, we overexpressed *Bmp8b* in wild-type osteoblasts followed by culture under osteogenic conditions. The differentiation determined by ALP staining (day7) and alizarin red staining (day14) showed that overexpression of BMP8B, in contrast with BMP2, had no evident effects on osteoblastogenesis. (Figure S3 A&B). Overall, these data suggest that the increased expression of *Wnt7b* and *Bmp2* may contribute to the enhanced osteoblast activity in *Lsd1*^*prx1*^ mice.

### *Wnt7b* and *Bmp2* are directly regulated by LSD1 in osteoblasts

**Fig. 5:**
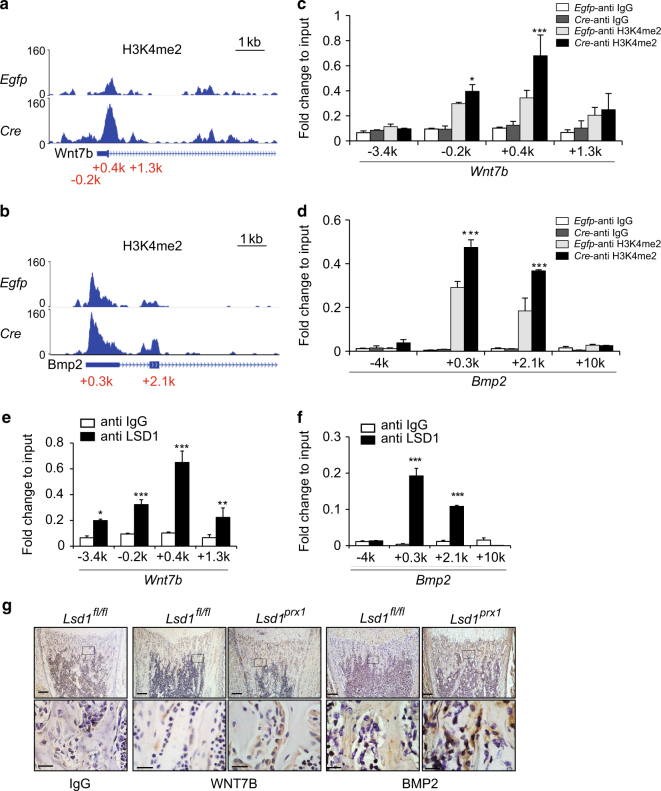
*Wnt7b* and *Bmp2* are directly regulated by LSD1 in osteoblasts. **a**, **b** Genomic tracks display ChIP-seq data using H3K4me2 across the promoter region of *Wnt7b* (**a**) and *Bmp2* (**b**). **c**, **d** ChIP-qPCR analysis of H3K4me2 enrichment in the *Wnt7b* (**c**) and *Bmp2* (**d**) promoter regions in calvarial cells. *n* = 3 for each group, all data represent means ± SD ANOVA followed by Tukey’s post hoc test was performed, *P < 0.05, ***P < 0.001. **e**, **f** ChIP-qPCR analysis of LSD1 enrichment in the *Wnt7b* (**e**) and *Bmp2* (**f**) promoter regions in calvarial cells. *n* = 3 for each group, all data represent means ± SD Unpaired *t-*test was performed, *P < 0.05, **P < 0.01, ***P < 0.001. **g** Immunohistochemistry for WNT7B and BMP2 showing increased expression in a coronal section of the proximal tibias of 1-week-old *Lsd1* ^*prx1*^ mice compared to littermate controls. The images are representative of three mice per group. Scale bar, upper panel: 200 μm, lower panel: 40 μm.

Our ChIP-seq data indicated that H3K4me2 marks are specifically enriched in the promoter region of the *Wnt7b* and *Bmp2* genes (Fig. 5a, b). ChIP-qPCR analysis confirmed H3K4me2 enrichment at the *Wnt7b* and *Bmp2* promoters in *Lsd1*-deficient cells (Fig. 5c, d). To examine whether LSD1 could directly influence H3K4me2 levels at the *Wnt7b* and *Bmp2* promoters, we performed ChIP-qPCR analysis using an LSD1-specific antibody and found that LSD1 directly bound to these regions (Fig. 5e, f). To investigate whether WNT7B and BMP2 protein levels were increased in vivo, we examined their expression in tibia bone which turned out that both proteins were upregulated in bone area of *Lsd1*^*prx1*^ mice compared with *Lsd1*^*fl/fl*^ littermate controls (Fig. 5g). Additional evidence for a functional role of BMP2 downstream of *Lsd1* deficiency stems from the forepaw phenotype of *Lsd1*^*Prx1*^ mice. The duplication of a regulatory element that leads to high expression level of BMP2 in the developing limb has been demonstrated to cause brachydactyly type A2 (BDA2), a malformation characterized by hypoplastic middle phalanges of the second and fifth fingers.^24^ Interestingly, we observed the same phenotype of hypoplastic middle phalanges of the second and fifth fingers in *Lsd1*^*prx1*^ mice, consistent with upregulated BMP2 expression in *Lsd1*-deficient mice (Figure S4 A&B). These data support the conclusion that LSD1 directly binds to the *Wnt7b* and *Bmp2* promoters and regulates their expression.

### WNT7B-related mTOR signaling and BMP2-related BMP signaling contribute to the increased bone mass in in *Lsd1*^*prx1*^ mice

**Fig. 6:**
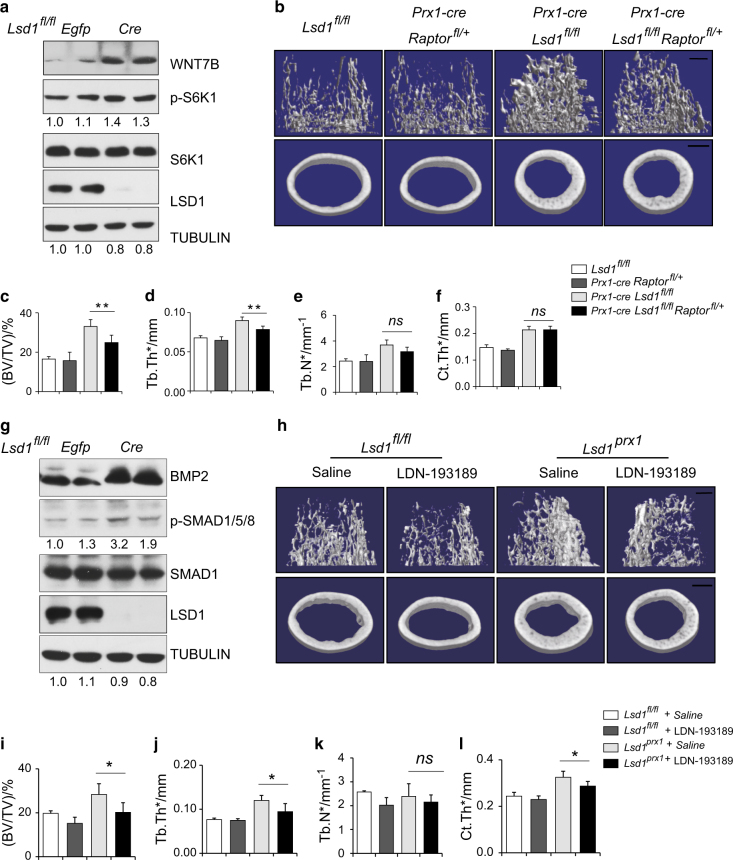
WNT7B related mTOR signaling and BMP2 related BMP signaling contribute to the increased bone mass in *Lsd1*^*prx1*^ mice. **a** *Lsd1* ^*fl/fl*^ calvarial cells derived from P3 mice were infected with Egfp or Cre virus followed by culture in osteoblast differentiation medium for 7 days. WNT7B and pS6K1 levels were measured by western blot. Results are representative of two independent experiments. **b**–**f** Femurs from 6 weeks female mice (*n* = 4–6) were analyzed by μCT. **b** 3D reconstructions of the trabecular bone and midshaft cortical bone. Quantitative parameters of trabecular bone (**c**–**e**) and cortical bone (**f**) were analyzed. ANOVA followed by Tukey’s post hoc test was performed. **P < 0.01. Scale bar, 0.5 mm. **g** *Lsd1* ^*fl/fl*^ calvarial cells derived from P3 mice were infected with Egfp or Cre virus followed by culture in osteoblast differentiation medium for 7 days. LSD1, p-SMAD1/5/8, and BMP2 levels were measured by western blot. Results are representative of two independent experiments. **h**–**l** Femurs from 9 weeks *Lsd1*^*prx1*^ and *Lsd1*^*fl/fl*^ male mice (*n* = 4–6) after BMP inhibitor LDN-193185 injection for 4 weeks were analyzed by μCT. **h** 3D reconstructions of the trabecular bone and midshaft cortical bone. Quantitative parameters of trabecular bone (**I**–**k**) and cortical bone (**l**) were analyzed. ANOVA followed by Tukey’s post hoc test was performed. *P < 0.05. Scale bar in **h**: 0.5 mm.

WNT7B has been reported to promote osteoblast differentiation in part through mTORC1 signaling rather than the canonical WNT signaling pathway^21^ and the mTOR inhibitor Rapamycin can inhibit osteoblast proliferation and differentiation.^25^ In agreement with this, we found WNT7B expression was increased in *Lsd1*-deficient osteoblasts along with increased phosphorylation of S6K1 protein (Fig. 6a) and treatment with Rapamycin for 14 days attenuated the enhanced osteoblast differentiation of *Lsd1*-deficient cultures, although not completely reversed (Figure S5 A&B). To investigate whether increased WNT7b-mTORC1 signaling in osteoblasts is responsible for the mice phenotype in vivo, we crossed the *Raptor*^*fl/fl*^ mice with *Prx1-cre, Lsd1*^*fl/fl*^ mice to block mTORC1 signaling in osteoblasts. Since *Prx1-cre Raptor*^*fl/fl*^ mice exhibits neonatal death,^26^ we collected 6-week-old *Prx1-cre, Raptor*^*fl/+*^ *Lsd1*^*fl/fl*^ and control mice for bone phenotype analysis. The *Raptor* mRNA level was decreased about 30% in long bone of *Prx1-cre Raptor*^*fl/+*^ mice (Figure S5 E). *Prx1-cre, Raptor*^*fl/+*^ trabecular bone mass are comparable to *Lsd1* ^*fl/fl*^ mice, while *Prx1-cre, Raptor*^*fl/+*^ *Lsd1*^*fl/fl*^ mice partially rescued the increased trabecular bone mass of *Prx1-cre, Lsd1*^*fl/fl*^ mice although the cortical bone phenotype were not recued (Fig. 6b–f). These data suggested that Wnt7b-mTORC1 signaling partially contributed to the increased bone mass of *Prx1-cre Lsd1*^*fl/fl*^ mice.

Likewise*, Lsd1* deletion in differentiating osteoblasts in vitro resulted in BMP2 upregulation (Fig. 6g). BMP2 promotes osteoblast differentiation through the canonical SMAD signaling pathway.^27^ Consistently, we observed increased phosphorylation of SMAD1/5/8 protein in *Lsd1* knockout osteoblasts (Fig. 6g). Next, we investigated whether inhibition of canonical SMAD signaling could reverse the enhanced osteoblast differentiation. *Lsd1* deficient and control calvarial osteoblast cultures were treated with LDN-193189, a BMP receptor kinase inhibitor, for 14 days. The enhanced differentiation observed in *Lsd1*-deficient osteoblasts was significantly attenuated upon LDN-193189 treatment (Figure S5 C&D). To examine the in vivo effect of LDN-193189, *Lsd1*^*prx1*^ mice and littermate controls were treated with LDN-193189 for 4 weeks and femur bone mass were measured by micro CT. Both trabecular and cortical bone phenotype of *Lsd1*^*Prx1*^ mice were attenuated after LDN-193189 treatment (Fig. 6h–l). These data suggest that elevated BMP2 levels activating the canonical SMAD signaling pathway is involved in high bone mass formation of *Lsd1*^*Prx1*^ mice.

### WNT7B and BMP2 promote osteoblast differentiation additively in *Lsd1* deficient osteoblasts

**Fig. 7:**
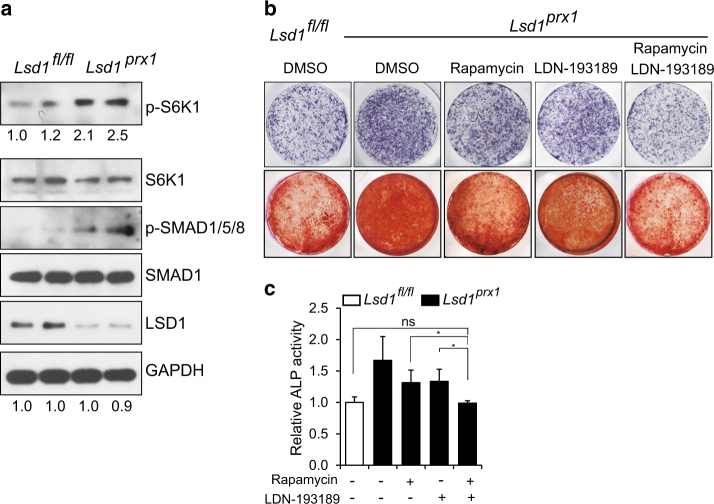
Inhibition of BMP and mTOR signaling reverses enhanced osteoblast differentiation in *Lsd1* deleted osteoblasts. **a** BMP and mTOR signaling protein levels were analyzed by western blot using primary calvarial bone samples from 4-week-old Ls*d1*^*prx1*^ and *Lsd1*^*fl/fl*^ mice. Results are representative of two independent experiments. **b**, **c** ALP staining (day7) and alizarin red staining (day 14) (**b**) and ALP quantification (**c**) of osteoblast precursors from *Lsd1*^*fl/fl*^ and *Lsd1* ^*prx1*^ mice treated with Rapamycin and LDN-193189 in osteogenic cultures for 14 days. Data in **c** are presented as mean ± s.d (*n* = 6). ANOVA followed by Tukey’s post hoc test was performed. *P < 0.05, **P < 0.01.

We next examined whether there was evidence of increased WNT7B and BMP2 downstream signaling in primary *Lsd1*-deficient mice. As shown in Fig. 7a, both p-S6K1 and p-SMAD1/5/8 levels were increased in calvarial bone samples from 4-week-old *Lsd1*^*prx1*^ mice compared with *Lsd1*^*fl/fl*^ littermate controls, consistent with increased WNT7B and BMP2 signaling activity. We then asked whether WNT7B and BMP2 act additively downstream of LSD1 deficiency to promote osteoblast differentiation. We generated lentiviral constructs to overexpress *Wnt7b* or/and *Bmp2* in wild-type osteoblasts followed by culture under osteogenic conditions and analyzed cell differentiation state using ALP staining at day7 and alizarin red staining at day14. As expected, both *Wnt7b* and *Bmp2* overexpression alone promoted osteoblast differentiation, which was further enhanced by co-expression of *Wnt7b* and *Bmp2* (Figure S6 A&B). Next, we investigated whether blocking BMP2 and WNT7B signaling together abrogated the enhanced differentiation in *Lsd1* null cells, the combination of Rapamycin and LDN-193289 but not the individual inhibitors completely reversed the enhanced differentiation of *Lsd1*^*prx1*^ osteoblasts back to wild-type levels (Fig. 7b, c). Taken together, our results indicate that LSD1 deficiency results in elevated WNT7B and BMP2 expression, which additively promote osteoblast differentiation and increased bone mass observed in *Lsd1*^*prx1*^ mice.

### LSD1 inhibitor TCP increased mice bone mass

**Fig. 8:**
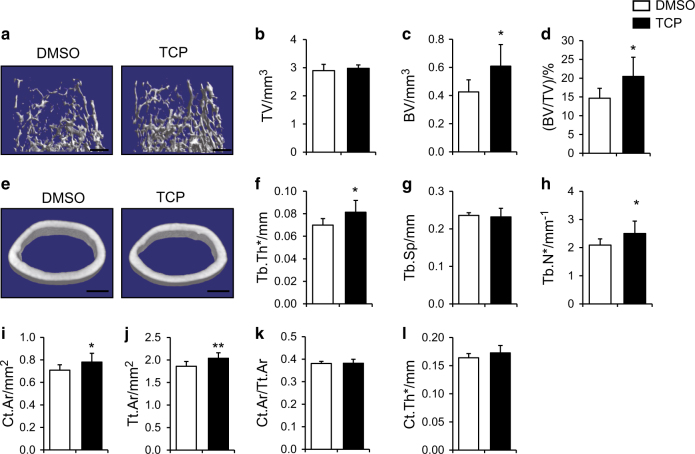
LSD1 inhibitor TCP increased mice bone mass. **a**–**l** Femurs from TCP-treated or DMSO-treated male mice were analyzed by μCT. 3D reconstructions of the trabecular bone (**a**) and midshaft cortical bone (**e**). Quantitative parameters of trabecular bone (**b**–**h**) and cortical bone (**i**–**l**) were analyzed, *n* = 7, statistical analysis, unpaired *t*-test, *P < 0.05, **P < 0.05. Scale bar in **a**, **e**: 0.5 mm.

Tranylcypromine (TCP) is a monoamine oxidase inhibitor that has been widely used to inhibit the activity of LSD1 protein. To test whether TCP could upregulate *Bmp2* and *Wnt7b* expression level, we treated calvairal osteoblast with 50 μM TCP for 48 h, the transcription level was measured by RT-PCR and we found that both *Bmp2* and *Wnt7b* mRNA level was increased upon TCP treatment (Figure S7 A). We next sought to identify whether TCP increase mice bone mass in vivo, we injected 5-week-old wild-type mice with 3 mg·kg^-1^ TCP or DMSO subcutaneous every other day for 1 month. The trabecular bone volume and trabecular bone thickness as well as trabecular number were increased after TCP treatment (Fig. 8a–l). These data suggested that TCP had anabolic effects on bone formation and highlighted the potential clinical value for osteoporosis treatment of LSD1 inhibitors.

## Discussion

Here we demonstrate that the histone H3K4 demethylase LSD1 has an important function in skeletal development in vivo to repress osteoblast activity and bone formation. We observed increased bone mass in mice lacking *Lsd1* selectively in mesenchymal progenitor cells and showed that this was due to enhanced osteoblastogenesis. These results are consistent with a previous in vitro study, which demonstrated increased osteogenesis of human adipose-derived stem cells by LSD1 inhibition.^18^ Genome-wide expression analysis revealed markedly increased expression of osteoblast-stimulating factors *Wnt7b* and *Bmp2* in LSD1 mutant osteoblasts and demonstrated that both of these factors stimulated osteoblastogenesis acting additively.

WNT ligands play a central role in bone development and homeostasis through β-catenin-dependent and -independent signaling pathways.^28–30^ In the β-catenin-dependent canonical WNT pathway, WNT binds to Frizzled receptors and the low-density lipoprotein receptor-related protein 5 or 6 (LRP5/6) that stabilizes cytosolic β-catenin and thereby stimulates transcription of downstream target genes. Wnt-β-catenin signaling is critical for osteoblast lineage commitment and promotes osteoblast precursor proliferation and differentiation.^31–34^ In the noncanonical WNT signaling pathway, WNT promotes bone formation through G-protein-linked PKCδ activation,^30^ and WNT7B activates mTORC1 to increase osteoblast differentiation.^21^ Targeted induction of *Wnt7b* in the osteoblast lineage resulted in profound bone mass accrual.^21^ Our study found that LSD1 acted as an upstream factor regulating *Wnt7b* expression in osteoblasts. Since WNT7B can induce other signaling pathways like mTORC2 and PKCδ,^21,30^ it is possible that these pathways may also be involved in the augmented osteoblast activity in *Lsd1*^*prx1*^ mice, a possibility which needs further investigation.

BMPs are a large family of morphogens, some of which, including BMP2, promote bone formation and increase osteoblastogenesis in vitro.^35^ Aberrant BMP signaling has been identified in several human skeletal diseases, for instance, mutations in the bone morphogenetic protein receptor 1b (BMPR1B) or its ligand growth and differentiation factor 5 (GDF5) lead to autosomal-dominant brachydactyly type A2 (BDA2),^36^ and duplication of a regulatory enhancer that increases the expression of *Bmp2* in the developing limb also causes BDA2.^24^ Our finding that mice lacking *Lsd1* in limbs have a brachydactyly phenotype resembling BDA2 (Figure S4 A&B) thus provides supportive evidence that *Lsd1* deficiency results in increased *Bmp2* expression in vivo.

In this study, we found inhibition of LSD1 could regulate WNT7B and BMP2 to promote osteoblast differentiation. However, many other genes were also upregulated upon LSD1 deletion in osteoblast progenitors. Some of them are also associated with osteoblast differentiation, such as *Pdgfb* and *Hoxa10*,^37,38^ while most of them have unknown function in osteoblast. Further investigation about the functions of other LSD1-regulated genes will be needed to reveal the complex role of LSD1 in skeletal development.

The higher bone mass and greater bone strength in *Lsd1*^*prx1*^ mice was due to increased bone formation, as bone resorption in *Lsd1*^*prx1*^ mice is increased rather than impaired. This enhanced osteoclast activity may result from attempted coordination of osteoblast and osteoclast function in vivo to achieve balanced bone remodeling. Several LSD1 inhibitors such as GSK2879552, GSK-LSD1, TCP, Pargyline and some tranylcypromine analogs^14–17,39^ have been identified and used to effectively inhibit LSD1 activity. In line with our results that LSD1 conditional knockout mice displayed increased bone mass, recently, Lv et al. found that LSD1 inhibitor Pargyline can improve osteoblast differentiation and partially rescue the osteoporotic conditions in aged or ovariectomized mouse models.^40^ Consistent with this, we found that TCP can also increase mice bone mass. The effects of TCP are even stronger than Pargyline as TCP can improve the bone mass of young mice while Pargyline has little effects.^40^ Considering the wide range of LSD1 functions in many systems and cells, direct osteoblast-specific delivery system for LSD1 inhibitors is needed to ensure the safety and efficacy when it is used for the treatment of bone-related diseases. Currently, several osteoblast targeting delivery systems have been developed for drug delivery, such as aptamer-functionalized lipid nanoparticles and SDSSD-modified polyurethane nanomicelles.^41,42^ These may pave the way for LSD1 inhibitors to specifically target osteoblasts.

Overall, our study illustrates an unexpected function for the epigenetic regulator LSD1 in osteoblast differentiation, providing a potentially attractive targeted therapy for diseases of low bone mass.

## Methods

### Mice

*Lsd1*^*fl/fl*^ mice^43^ bearing loxP sites flanking exons 6 of the *Lsd1* gene were kindly provided by Dr. Michael Rosenfeld. *Prx-1-Cre mice*^44^ and *Raptor*^*fl/fl*^ mice were purchased from the Jackson Laboratory. *Lsd1*^*fl/fl*^ mice were cross-bred with *Prx1-Cre* mice to specifically delete *Lsd1* expression in mesenchymal cells. All mice were bred and maintained under Specific Pathogen Free (SPF) conditions in the institutional animal facility of the Shanghai Institute of Biochemistry and Cell Biology, Chinese Academy of Sciences. Age- and sex-matched littermates were used as control mice.

### Cell culture

hMSCs were purchased from Cyagen. Primary cultures of murine osteoblasts were isolated from calvariae of 4- to 6-day-old *Lsd1*^*prx1*^ mice and *Lsd1*^*fl/fl*^ littermates. Primary BMSCs were isolated from the long bone of mice, bone marrow was flushed with PBS and then pelleted at 1 200 r·min^-1^ for 4 min, the pellet was suspended in α-MEM with 10% FBS and cultured. Osteoblast differentiation was induced by culture in osteogenic media containing 50 μg·mL^-1^ ascorbic acid (Sigma, A5960) and 5 mmol·L^-1^ β-glycerophosphate (Sigma, G9422). For alizarin red staining, cells were fixed at day 14 of culture with 10% neutral formalin buffer and stained with alizarin red staining buffer (Cyagen) for 15 min, the mineralized area was determined using Image J program. For ALP assay, cells were fixed on day 7 with 10% neutral buffered formalin and subjected to ALP staining. For quantification of ALP activity, osteoblasts were incubated with Alamar Blue (Sigma-Aldrich) for 4 h. After cellularity was measured at 580 nm, supernatants were removed and cells incubated with 6.5 mmol·L^-1^ Na_2_CO_3_, 18.5 mmol·L^-1^ NaHCO_3_, 2 mmol·L^-1^ MgCl_2_, and phosphatase substrate (Sigma-Aldrich) for 30 min, ALP activity was then read with a luminometer at 405 nm. Rapamycin (Sigma) was used at a concentration of 10 nmol·L^-1^. LDN-193189 (Selleck) was used at a concentration of 10 nmol·L^-1^. Tranylcypromine hydrochloride (TCP) [TOCRIS (3852)] was used at a concentration of 50 μmol·L^-1^ in in vitro cell culture.

### ChIP assays

Primary calvarial cells isolated from *Lsd1*^*fl/fl*^ mice were infected with *Egfp*- or *Cre*-expressing lentivirus.^45^ Cells (1 × 10^6^) were used for each immunoprecipitation as described previously.^23^ Briefly, cells were crosslinked with 1% formaldehyde at room temperature for 10 min. Glycine with a final of 125 mmol·L^-1^ was added to quench the crosslinking. Cells were scraped, washed by PBS three times and lysed with SDS buffer (1%SDS, 10 nmol·L^-1^ EDTA, 50 mmol·L^-1^ Tris). Samples were then sonicated to produce 0.2–0.7 kb DNA fragments. Eight micrograms indicated antibody were used for immunoprecipitation overnight at 4 °C. Protein G beads were then added and incubated for 2 h to isolate antibody-bound chromatin. The ChIP DNA was purified with PCR purification kit (Qiagen) and quantified by real-time PCR.

### Antibodies and reagents

Anti-BMP2 antibody (ab14923), anti-LSD1 (ab17721), and anti-WNT7B (ab94915) were obtained from Abcam. Anti-Histone H3 (A2348), anti-H3K4me1 (A2355), anti-H3K4me2 (A2356), and anti-H3K4me3 (A2357) were purchased from ABclonal Technology. Anti-TUBULIN antibody (SC-23948) was from Santa Cruz Biotechnology. Anti-SMAD1 (6944), anti-phospho-SMAD1/5/9 (13820), anti-H3K9me1(14186), anti-H3K9me2(4658), anti-H3K9me3(14186), and anti-pS6K1(9234) were purchased from Cell Signaling Technology.

### Vector construction

All shRNAs were cloned into pLKO.1 vector, the shRNA target sequences are listed in Supplementary Table S3.

### Extracting bone protein

Four-week-old *Lsd1*^*prx1*^ mice and *Lsd1*^*fl/fl*^ littermate controls were sacrificed, skin and muscle were removed and parietal bones or long bones (tibias and femurs) were harvested. Bones were placed in 2 mL cryovial and submerged in liquid nitrogen to freeze. Bones were then removed from cryovial, wrapped in aluminum foil, and pulverized with hammer. Bone fragments were transferred into cell lysis buffer and homogenized by homogenizer. Mixtures were centrifuged at 15 000 r·min^-1^ for 20 min at 4 °C, and supernatant was collected.

### Histology and immunostaining

Tissues were fixed in 4% paraformaldehyde for 48 h and incubated in 15% DEPC-EDTA (pH 7.8) for decalcification. Then specimens were embedded in paraffin and sectioned at 5 μm. Immunohistochemistry was performed using TSA-biotin amplification system (Perkin Elmer Life Sciences) according to the manufacturer’s instructions using antibody against BMP2 or WNT7B. The proportion of positive cells in each field was determined using Image J program. Digoxigenin (DIG)-labeled RNA probes were generated according to the manufacturer’s protocol. Briefly, DIG-labeled antisense probes were generated to detect *Col1a1*, *Ocn* mRNA expression. Probes were then hybridized with paraffin sections and visualized using an anti-DIG HRP conjugate system.

### MicroCT analysis

The mouse hindlimbs and skulls were skinned and fixed in 70% ethanol. Femurs were scanned using a Skyscan 1176 scanner (Bruker, Kartuizersweg, Belgium) with a spatial resolution of 8.96 μm. The X-ray energy is 70 kVp and 305 μA.

To analyze trabecular bone, a region of 2.0 mm of the distal metaphysis starting 0.7 mm from the proximal end of the distal femoral growth plate and a threshold of 75–255 permille were used. To analyze cortical bone, a region of 0.5 mm of the femoral cortical bone starting 3.7 mm from the proximal end of the distal femoral growth plate and a threshold of 125–255 permille were used. A Gaussian noise filter optimized for murine bone was applied to reduce noise in the thresholded 2D image. Three-dimensional reconstructions were created by stacking the two-dimensional images from the contoured regions. Indices of trabecular and cortical bone were shown according to the guidelines.^46^ Skulls were scanned using a Skyscan 1172 scanner (Bruker, Kartuizersweg, Belgium) with a spatial resolution of 9 μm. The X-ray energy is 49 kVp and 200 μA. Cylindrical regions of interest (ROIs) with a diameter of 3.5 mm in parietal bone and a threshold of 80–255 permille were used. A Gaussian noise filteroptimized for murine bone was applied to reduce noise in the thresholded 2D image.

### Quantum GX microCT analysis

To analyze the fat store of *Lsd1*^*prx1*^ and *Lsd1*^*fl/fl*^ mice, 4-week-old female mice were anesthetized and scanned by Quantum GX microCT (PerkinElmer, Waltham, MA, USA) with a spatial resolution of 72 μm, view-imaging at 36 × 36 mm. The X-ray energy is 70 kVp and 114 μA. Subcutaneous and visceral fat volumes were analyzed.

### RNA-seq

Total RNA was isolated using TRIZol (Sigma) from the egfp or cre virus-infected *Lsd1*^*fl/fl*^ calvarial cells. cDNA library preparation and sequencing was performed according to the Illumina’s standard protocol.

### Real-time RT-PCR analysis

Total RNA was prepared using TRIzol (Sigma) and was reverse transcribed into cDNA with the PrimeScript™ RT Reagent Kit (TakaRa). Real-time quantitative PCR was performed with the BioRad CFX96 system. The sequences of oligonucleotides used for quantitative PCR (qPCR) are listed in Supplementary Table S2.

### Three-point bending test

Four-week-old male mice femurs were collected and stored in 70% ethanol. Strength tests were performed with Instron 3345 at the right femur midshaft with a displacement rate of 0.03 mm·s^-1^ until the bone fractured; span length was 6 mm. Maximum load (a measurement of the maximum force that the bone withstood before fracture) was determined using load-deflection diagrams.

### In vivo bone formation rate measurement

Four-week-old animals were injected intraperitoneally with calcein (20 mg·kg^-1^ body weight) and alizarin red S (50 mg·kg^-1^ body weight) on days −13 and −3 prior to tissue collection. Tibias were fixed in 70% (vol/vol) ethanol overnight, embedded in methyl methacrylate, and sectioned at 10 μm. Images were obtained using a fluorescence confocal microscope. Mineral apposition rate (MAR) in μm·d^-1^ and bone formation rate (BFR) were calculated from fluorochrome double labels at periosteal and endocortical surfaces.

### Serum measurements

Blood samples were collected from 5-week-old *Lsd1*^*prx1*^ and control mice. Serum OTEOCALIN and CTX-1 were measured by using commercially available ELISA kits (Shanghai Suer Biological technology) according to the instructions of manufacturer.

### In vivo TCP treatment

Five-week-old male C57BL6 mice were randomly divided into two groups. We injected these mice with either 3 mg·kg^-1^ TCP or DMSO subcutaneous every other day. One month after injection, we analyzed the bone mass of femurs of these mice with microCT.

### In vivo LDN-193185 treatment

Five-week-old *Lsd1*^*prx1*^ mice and *Lsd1*^*fl/fl*^ littermate controls were randomly divided into two groups separately. We injected these mice with either 3 mg·kg^-1^ LDN-193185 or saline i.p. every other day. Four weeks after injection, we analyzed the bone mass of femurs of these mice with microCT.

### Statistics

All results are presented as the mean ± SD Comparisons between two groups were analyzed using two-tailed, unpaired Student’s *t*-test. ANOVA followed by Tukey’s post hoc test was used when the data involve multiple group comparisons.

### Study approval

All experiments were performed according to the protocol approved by the Animal Care and Use Committee of Institute of Biochemistry and Cell Biology, SIBS, CAS.

## Electronic supplementary material

Supplementary information 1

Supplementary information 2
