# Supplementary material for: Histone demethylase LSD1 regulates bone mass by controlling WNT7B and BMP2 signaling in osteoblasts
Source: Bone Res. 2018 Apr 26;6:14. doi: 10.1038/s41413-018-0015-x (PMC5916912; doi:10.1038/s41413-018-0015-x)
Supplement: Supplementary file 1 — Supplementary information 1 [file 41413_2018_15_MOESM1_ESM.docx]

**Figure S1.** (A) Western Blot shows the deletion of LSD1 in long bone tissue of *Lsd1* *^prx1^* mice. (B)Representative photograph images of 4-week-old male *Lsd1* *^prx1^* and *Lsd1* *^fl/fl^* mice, bar: 1cm. (C) Body length of 4-week-old *Lsd1* *^prx1^* and *Lsd1* *^fl/fl^* mice, Data present mean±s.d (n=10). Statistical analysis, unpaired t-test, ****P*<0.001. (D) Body weight of *Lsd1* *^prx1^* and *Lsd1* *^fl/fl^* mice, Data present mean±s.d (n=10). ****P*<0.001. (E-G) Body fat stores of 4 weeks female *Lsd1* *^prx1^* and *Lsd1* *^fl/fl^* mice were analyzed by Quantum GX μCT. 3D reconstructions of the body fat (yellow) and Quantitative parameters of adipose tissue volume were analyzed (F&G), n=3-4, Statistical analysis, unpaired t-test. (H) Safranine O staining showed the embryonic endochondral bone formation of *Lsd1 ^prx1^* and *Lsd1 ^fl/fl^* mice, bar: 200um.

**Figure S2.** (A-L) *6* weeks female *Lsd1* *^prx1^* and *Lsd1* *^fl/fl^* mice were analyzed by μCT. 3D reconstructions of the trabecular bone (A) and midshaft cortical bone (H). Quantitative parameters of trabecular bone (B-G) and cortical bone(I-L) were analyzed, bone volume(BV), total volume(TV), trabecular number per cubic millimeter (Tb.N), trabecular thickness (Tb.Th), trabecular separation (Tb.Sp), cortical thickness (C.Th), Tt.Ar(total cross-sectional area), and Ct.Ar(cortical bone area), n=4-6, Statistical analysis, unpaired t-test, scale bar: 0.5mm. (M-O) Skulls of *6* weeks male *Lsd1* *^prx1^* and *Lsd1* *^fl/fl^* mice were analyzed by μCT. 3D reconstructions of the skull bone (M). BMD(bone mineral density)(N) and BV/TV(O) were analyzed. n=5, Statistical analysis, unpaired t-test, **P*<0.05,***P*<0.01. Scale bar: 1mm. (P-T) H&E staining of tibia of 4-week-old male *Lsd1* *^fl/fl^* and *Lsd1* *^prx1^* mice and histomorphometric analysis showed increased bone mass in *Lsd1^prx1^* mice. n=4, Statistical analysis, unpaired t-test, **P*<0.05, ***P*<0.01. Scale bar in P: 0.5mm.

**Figure S3.** (A&B) ALP staining (day7) and alizarin red staining (day 14)(A) and ALP quantification (B) of osteoblast precursors from WT mice infected with lentivirus expressing *Egfp*, *Bmp2* or *Bmp8b* virus in osteogenic cultures for 14 days. The data represent means±s.d (n=5). Statistical analysis, unpaired t-test, ****P*<0.001.

**Figure S4.** (A&B) Gross picture (A) and X-ray (B) image of skeletal preparations of the forepaw of 4-week-old *Lsd1* *^prx1^* mice and littermate controls. The images are representative of five mice per group. Scale bar, A&B:1mm.

**Figure S5.** (A&B) ALP staining (day7) and alizarin red staining (day 14) (A) and ALP quantification (B) of osteoblasts from *Lsd1^fl/fl^* mice infected with *Egfp* or *Cre* virus then treated with Rapamycin (10nM) in osteogenic cultures for 14 days. The data represent means±s.d (n=5). ANOVA followed by Tukey's post-hoc test was performed. *P<0.05, **P<0.01. (C&D) ALP staining (day7) and alizarin red staining (day 14) (C) and ALP quantification (D) of osteoblasts from *Lsd1^fl/fl^* mice infected with *Egfp* or *Cre* virus then treated with LDN-193189 (10nM) in osteogenic cultures for 14 days. The data represent means±s.d (n=5). ANOVA followed by Tukey's post-hoc test was performed. *P<0.05, **P<0.01, ***P<0.001. (E) RT-PCR analysis of *Raptor* expression in long bone tissue of *Prx1-cre Raptor ^fl/+^* and control mice. n=3, unpaired t-test, **P*<0.05.

**Figure S6.** (A&B) ALP staining (day7) and alizarin red staining (day 14)(A) and ALP quantification (B) of osteoblast precursors from WT mice infected with lentivirus expressing *Egfp*, *Bmp2* or/and *Wnt7b* virus in osteogenic cultures for 14 days. The data represent means±s.d (n=6). ANOVA followed by Tukey's post-hoc test was performed. *P<0.05, ***P*<0.01, ****P*<0.01.

**Figure S7.** (A) RT-PCR analysis of *Bmp2, Wnt7b* and *Lsd1* expression in calvarial osteoblast upon TCP treatment for 48h. n=3, unpaired t-test, ***P*<0.01.
