# Supplementary material for: Histone demethylase LSD1 regulates bone mass by controlling WNT7B and BMP2 signaling in osteoblasts
Source: Bone Res. 2018 Apr 26;6:14. doi: 10.1038/s41413-018-0015-x (PMC5916912; doi:10.1038/s41413-018-0015-x)
Supplement: Supplementary file 2 — Supplementary information 2 [file 41413_2018_15_MOESM2_ESM.pdf]

Figure S1

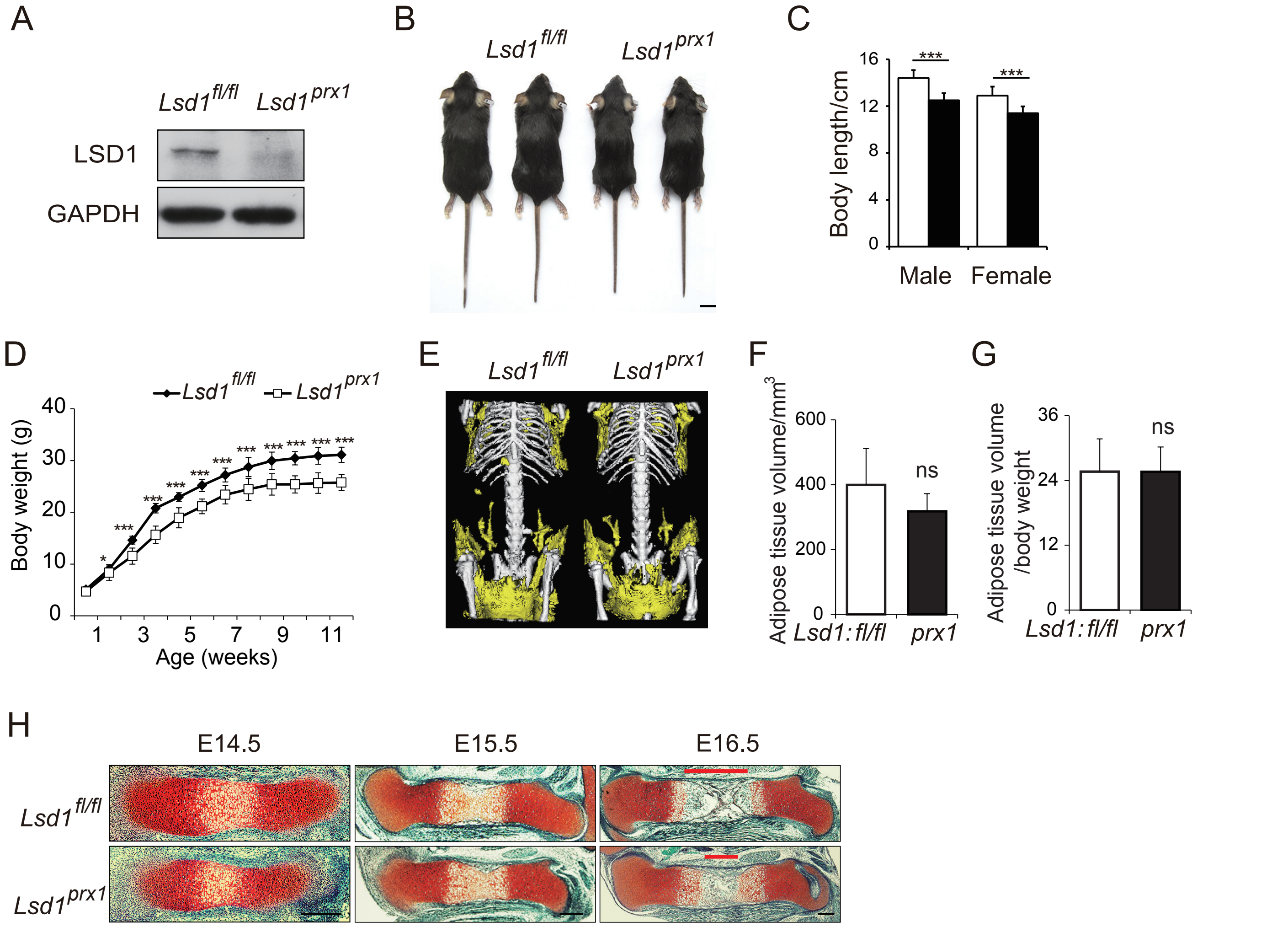
A
Lsd1 fl/fl
Lsd1 prx1
LSD1
GAPDH
B
Lsd1 fl/fl
Lsd1 prx1
C
Body length/cm
16
12
8
4
0
***
***
Male
Female
D
Lsd1 fl/fl
Lsd1 prx1
Body weight (g)
40
30
20
10
0
1
3
5
7
9
11
Age (weeks)
*
***
E
Lsd1 fl/fl
Lsd1 prx1
F
Adipose tissue volume/mm3
600
400
200
0
ns
Lsd1: fl/fl
prx1
G
Adipose tissue volume /body weight
36
24
12
0
ns
Lsd1: fl/fl
prx1
H
E14.5
E15.5
E16.5
Lsd1 fl/fl
Lsd1 prx1

Figure S2

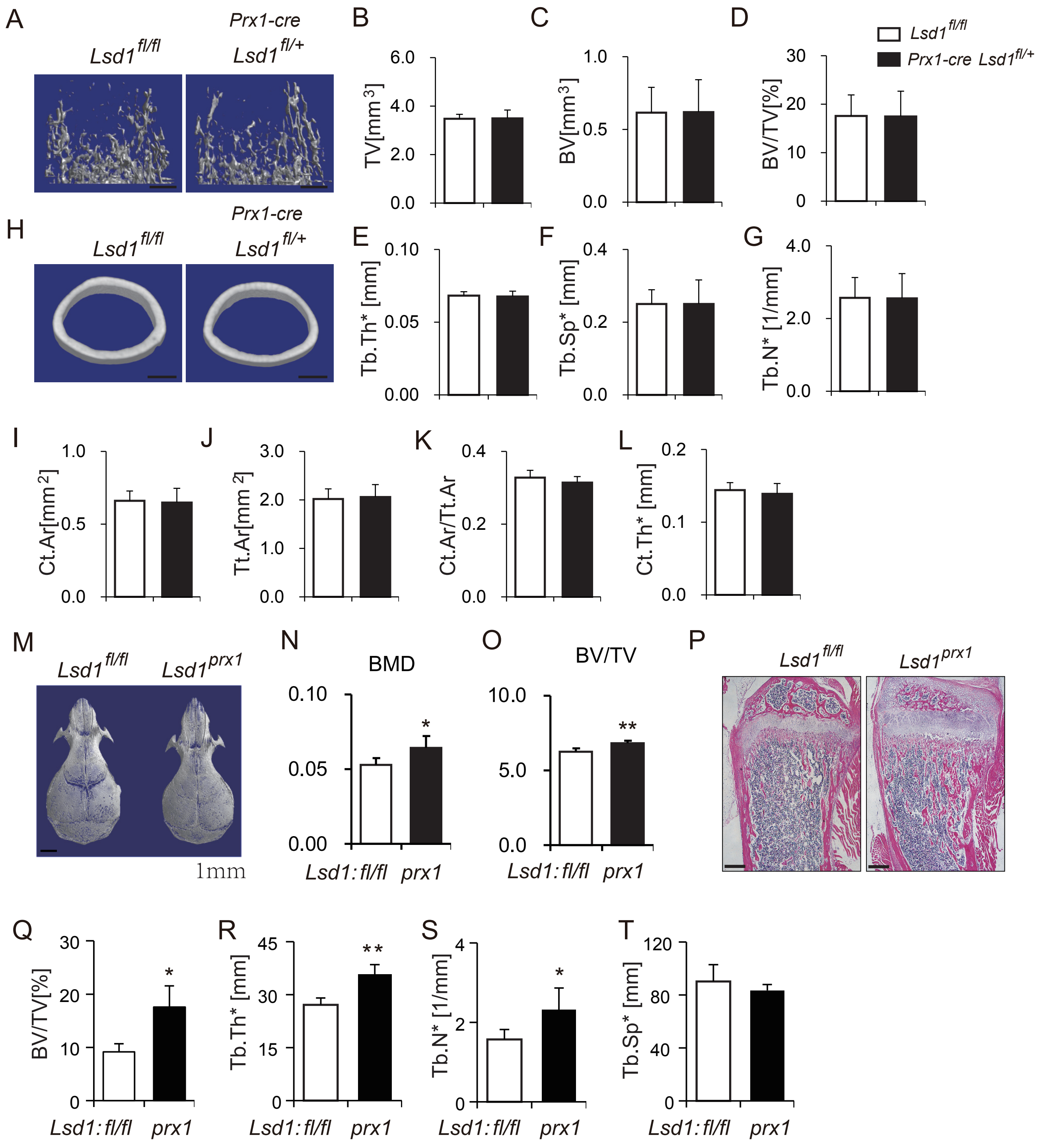
A
Lsd1 fl/fl
Prx1-cre Lsd1 fl/+
B
C
D
Lsd1 fl/fl
Prx1-cre Lsd1 fl/+
TV[mm3]
BV[mm3]
BV/TV[%]
H
E
F
G
Tb.Th* [mm]
Tb.Sp* [mm]
Tb.N* [1/mm]
I
J
K
L
Ct.Ar[mm2]
Tt.Ar[mm 2]
Ct.Ar/Tt.Ar
Ct.Th* [mm]
M
Lsd1 fl/fl
Lsd1 prx1
1mm
N
BMD
O
BV/TV
P
Lsd1:fl/fl prx1
Q
R
S
T
BV/TV[%]
Tb.Th* [mm]
Tb.N* [1/mm]
Tb.Sp* [mm]

Figure S3

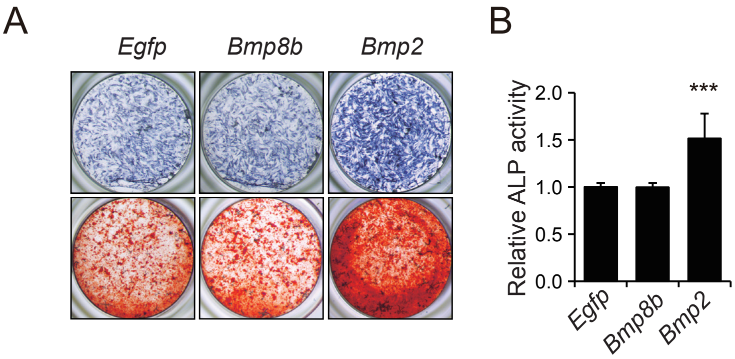
A
Egfp
Bmp8b
Bmp2
B
Relative ALP activity
2.0
1.5
1.0
0.5
0.0
***
Egfp
Bmp8b
Bmp2

Figure S4

A

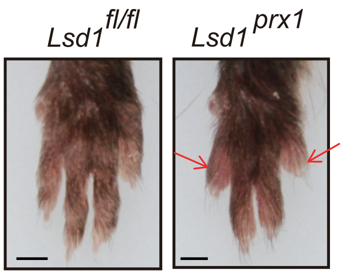

B

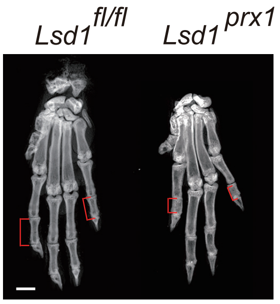

Figure S5

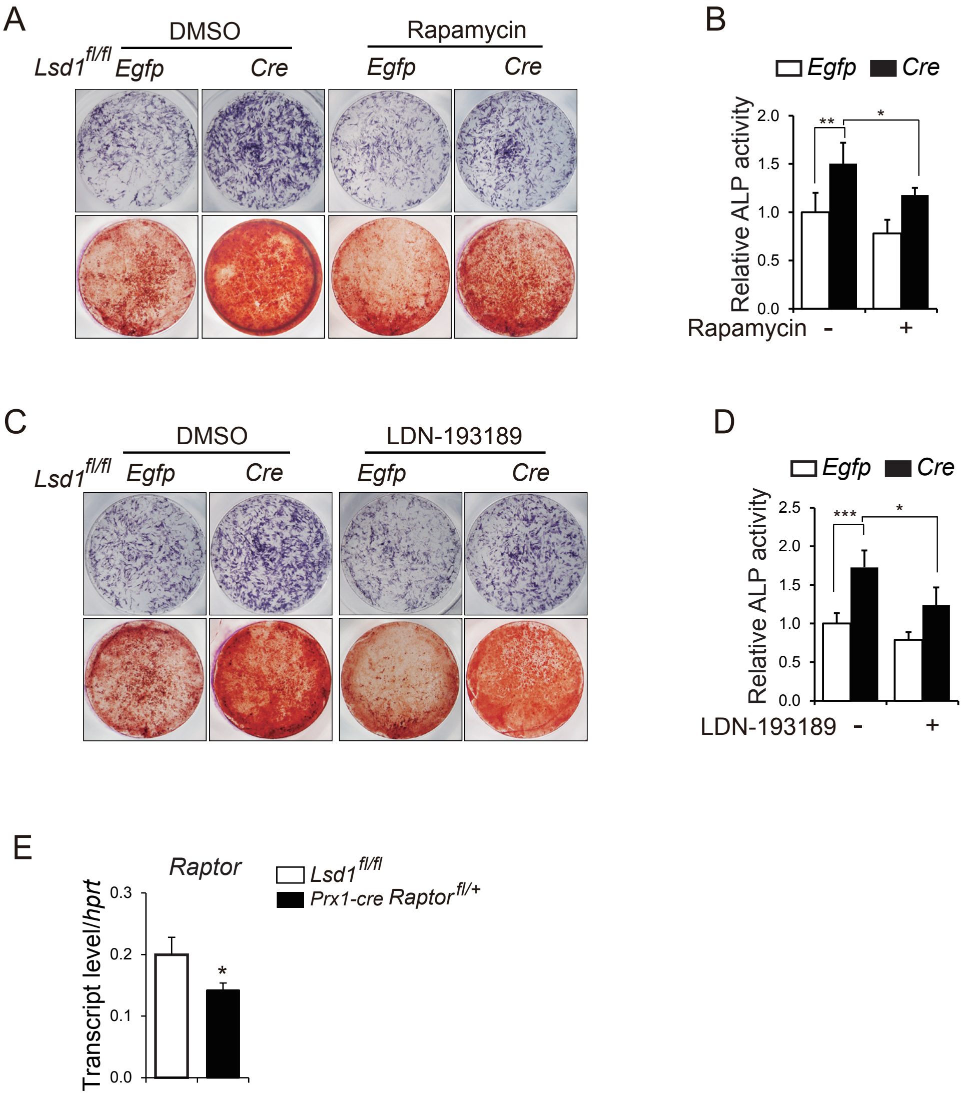
A
DMSO
Rapamycin
Lsd1 fl/fl
Egfp
Cre
Egfp
Cre
B
Egfp
Cre
Relative ALP activity
2.0
1.5
1.0
0.5
0.0
**
*
Rapamycin
-
+
C
DMSO
LDN-193189
Lsd1 fl/fl
Egfp
Cre
Egfp
Cre
D
Egfp
Cre
Relative ALP activity
2.5
2.0
1.5
1.0
0.5
0.0
***
*
LDN-193189
-
+
E
Raptor
Lsd1 fl/fl
Prx1-cre Raptor fl/+
Transcript level/hprt
0.3
0.2
0.1
0.0
*

Figure S6

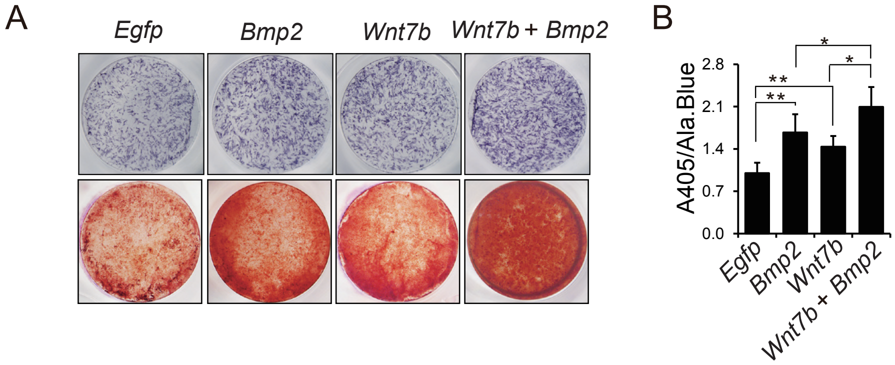
A
Egfp
Bmp2
Wnt7b
Wnt7b + Bmp2
B
A405/Ala.Blue
2.8
2.1
1.4
0.7
0.0
**
**
*
*
Egfp
Bmp2
Wnt7b
Wnt7b + Bmp2

Figure S7

A

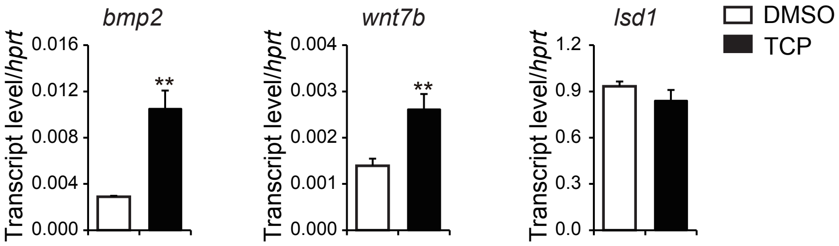

bmp2
wnt7b
lsd1
DMSO
TCP
Transcript level/hprt
0.016
0.012
0.008
0.004
0.000
**
0.004
0.003
0.002
0.001
0.000
**
1.2
0.9
0.6
0.3
0.0

Table S1. 114 genes with >2.0 fold increase of mRNA expression and >2.0 fold increase of H3K4me2 occupancy at TSS sites in LSD1 deficient cells.

No.	Gene	Egfp	Cre	Fold change
1	Cldn3	0.075	8.621	115.408
2	Bmp8b	0.029	3.231	112.247
3	Vat1l	0.023	2.200	96.437
4	Scn5a	0.058	3.919	67.161
5	Ccdc88c	0.013	0.817	63.237
6	Miat	0.018	1.051	57.026
7	Grhl3	0.031	1.548	50.590
8	Tjp3	0.029	1.423	49.505
9	Prph	0.101	4.616	45.717
10	D10Bwg1379e	0.039	1.784	45.452
11	Igsf1	0.102	4.428	43.536
12	Marveld3	0.035	1.320	37.634
13	Gap43	0.216	7.409	34.254
14	Ttbk1	0.023	0.718	30.828
15	Map7	0.086	2.579	30.054
16	Rasal1	0.026	0.617	23.764
17	Dlx3	0.033	0.727	22.133
18	Slc25a23	0.972	21.521	22.133
19	Atp2a3	0.055	1.182	21.611
20	Podxl	0.292	6.283	21.551
21	Atcay	0.023	0.466	20.552
22	Bspry	0.051	0.973	18.971
23	Adap1	0.718	12.943	18.023
24	Clgn	0.076	1.262	16.600
25	Kcnh3	0.047	0.737	15.809
26	Zfyve28	0.021	0.330	15.809
27	Ccdc184	0.037	0.586	15.809
28	Nat8l	0.050	0.767	15.414
29	Bex4	0.413	6.095	14.755
30	Ttc9	0.429	6.173	14.387
31	Mapk10	0.022	0.320	14.223
32	Unc13b	0.310	4.347	14.044
33	Cox7a1	1.014	13.628	13.438
34	Nrarp	0.066	0.893	13.438
35	Lamc3	0.027	0.347	12.647
36	Rtn4rl2	0.448	5.433	12.121
37	Trank1	0.030	0.361	11.857
38	Gjb3	0.914	10.715	11.726
39	Abcg1	0.098	1.085	11.067
40	Tnfsf15	0.042	0.464	11.067
41	Dsp	0.201	2.224	11.067
42	Slc52a3	0.326	3.603	11.042
43	Perp	3.908	42.876	10.972
44	Mtmr7	0.510	5.452	10.690
45	Bex1	0.720	7.020	9.749
46	Krt18	0.463	4.493	9.711
47	Fmnl1	0.375	3.590	9.579
48	Gpr4	0.029	0.279	9.486
49	Notum	0.913	7.695	8.432
50	Pnp2	0.466	3.929	8.432
51	Kif1a	0.655	5.179	7.905
52	Wnt7b	0.174	1.357	7.801
53	Pdgfb	0.633	4.502	7.114
54	Slc4a8	0.109	0.761	7.015
55	Selp	0.316	2.194	6.932
56	Rassf10	0.096	0.643	6.719
57	Adora2a	0.164	1.090	6.640
58	Celsr2	0.076	0.487	6.412
59	Lmtk3	0.611	3.917	6.411
60	Matk	0.096	0.608	6.325
61	Arhgef16	0.219	1.388	6.324
62	Jag2	0.123	0.776	6.324
63	Irf8	0.328	2.028	6.180
64	Jakmip2	0.571	3.462	6.060
65	Tcfl5	1.653	9.272	5.609
66	Fgf16	0.056	0.307	5.533
67	Cbln1	0.148	0.819	5.533
68	Ccdc64	0.383	2.043	5.336
69	Rims2	0.368	1.892	5.144
70	Cdh13	0.151	0.777	5.138
71	Bend4	0.447	2.267	5.066
72	Slc2a6	5.510	27.230	4.942
73	Rab6b	0.278	1.345	4.842
74	Dmpk	13.815	66.571	4.819
75	Gm11837	0.344	1.633	4.743
76	Hoxc13	0.071	0.335	4.743
77	Map3k15	0.494	2.342	4.743
78	Dmc1	0.117	0.557	4.743
79	Msln	9.120	42.926	4.707
80	Nacad	0.403	1.724	4.282
81	Doc2b	0.253	1.016	4.013
82	C77370	0.184	0.733	3.984
83	Slc35g1	3.085	11.848	3.841
84	Adam8	3.800	14.581	3.837
85	Ccdc3	0.161	0.609	3.794
86	Pstpip1	3.737	13.558	3.628
87	Chn1	1.207	4.367	3.617
88	Gpr162	1.180	4.234	3.589
89	Lypd1	0.171	0.610	3.557
90	Icam1	2.295	8.163	3.557
91	Dchs1	0.421	1.439	3.416
92	Rab39b	1.083	3.659	3.377
93	Slc16a3	2.007	6.723	3.349
94	Hoxa10	0.161	0.538	3.340
95	Whrn	0.368	1.226	3.334
96	Pacsin1	0.080	0.253	3.162
97	2010005H15Rik	0.496	1.570	3.162
98	Bmp2	0.094	0.296	3.162
99	Fsd1	0.920	2.910	3.162
100	Slain1	0.093	0.295	3.162
101	Gal	0.161	0.509	3.162
102	Cox6b2	8.380	25.444	3.036
103	Rasip1	0.267	0.802	3.004
104	Hrh1	0.198	0.591	2.987
105	Tmem30b	1.253	3.680	2.936
106	Necab1	0.100	0.290	2.898
107	Eml2	6.112	17.136	2.804
108	Arap3	0.627	1.585	2.530
109	C3	31.345	78.924	2.518
110	Itga10	4.489	11.236	2.503
111	Fam19a5	1.314	3.167	2.411
112	C1ql1	1.519	3.481	2.292
113	Robo3	0.557	1.265	2.273
114	Arhgap36	4.973	10.057	2.023

Table S2. Primers for Realtime-PCR analysis

species	primer name	forward sequence	reverse sequence
human	hLSD1	TCCTGGCCCCTCGATTC	ATGTTCTCCCGCAAAGAAGAGT
human	hGAPDH	TGGTATCGTGGAAGGACTCATGAC	ATGCCAGTGAGCTTCCCGTTCAGC
human	hALPL	ATCAGGGACATTGACGTGATC	TTCCAGGTGTCAACGAGGTC
human	hCol1a1	AGGGCCAAGACGAAGACATC	AGATCACGTCATCGCACAAC
mouse	mHPRT	GTTAAGCAGTACAGCCCCAAA	AGGGCATATCCAACAACAAACTT
mouse	mOsteocalcin	CTTGGTGCACACCTAGCAGA	CTCCCTCATGTGTTGTCCCT
mouse	mALP	CGGGACTGGTACTCGGATAA	ATTCCACGTCGGTTCTGTTC
mouse	mCol1a1	GCTCCTCTTAGGGGCCACT	CCACGTCTCACCATTGGGG
mouse	mOsterix	CCTTCCCTCACTCATTTCCTGG	TGTTGCCTGGACCTGGTGAGAT
mouse	mATF4	ATGGCGCTCTTCACGAAATC	ACTGGTCGAAGGGGTCATCAA
mouse	mRunx2	ATGCTTCATTCGCCTCACAAA	GCACTCACTGACTCGGTTGG
mouse	mOPN	CGAAGAAGCAGAAGTGGATG	GCTTCTTCTCCGTTGTCTCC
mouse	Cox6b2	CGGAAAGAGCACACAACCCT	GCAGCGCTCAAGTGCAAGGT
mouse	Cox7a1	GCTCTGGTCCGGTCTTTTAGC	GTACTGGGAGGTCATTGTCGG
mouse	Dlx3	CACTGACCTGGGCTATTACAGC	GAGATTGAACTGGTGGTGGTAG
mouse	Dmpk	CTGCTCGACCTTCTCCTGG	CACGCCCGATCACCTTCAA
mouse	Fgf16	GTGTTTTCCGGGAACAGTTTGA	GGTGAGCCGTCTTTATTCAGG
mouse	Grhl3	CCCGGCAAGACCAATACCG	AACCCCATGAATGCTCTCAAAT
mouse	Hoxa10	CCTGCCGCGAACTCCTTTT	GGCGCTTCATTACGCTTGC
mouse	Irf8	CGGGGCTGATCTGGGAAAAT	CACAGCGTAACCTCGTCTTC
mouse	Jag2	CAATGACACCACTCCAGATGAG	GGCCAAAGAAGTCGTTGCG
mouse	Kcnh3	CTGTGACCTCACGGGTTTCTC	GGGCCTTTCGGATCTGTTGG
mouse	Lmtk3	CCCTGGAACAGCGCAAGTT	CCGAAGGTATCGCTTCAGGTC
mouse	Map3k15	CCATGTGCTGACTATTTTTGCTG	TCTTTCGCCAGTTCATCACCT
mouse	Mapk10	AAGCCAGGGATTTGTTGTCTAAG	GGATGGAGGGAGACTCTCACT
mouse	Matk	CTTGTGAGGGAATCAGCTCGT	GGTGCCCATCTCGATGCAAA
mouse	Pdgfb	CATCCGCTCCTTTGATGATCTT	GTGCTCGGGTCATGTTCAAGT
mouse	Scn5a	ATGGCAAACTTCCTGTTACCTC	CCACGGGCTTGTTTTTCAGC
mouse	Slc2a6	AACCGAGGGACTCGACTATGA	CAAGGCATACCCAAAGCTGAA
mouse	Slc4a8	CTGCCTCATCAACGGCTCTG	GCTTCCCGCACTTTAACCCT
mouse	Tcfl5	CCAGCAACAAAACAAATGTACGA	TGGAGCAGGCACTTGTAAACA
mouse	Tnfsf15	TCTGGTCAGAAGGGATCAGAAG	GTCCTGCGAGGATGGGAAATG
mouse	Ttbk1	AAGGACGAAACCAACATGAGTG	GCGATCTTTGACCACGTAGTT
mouse	Wnt7b	TTTGGCGTCCTCTACGTGAAG	CCCCGATCACAATGATGGCA
mouse	hoxc13	GCCGTCTACACGGACATCC	CCCCAAATGGGTAACCATAGC
mouse	Slc16a3	TCACGGGTTTCTCCTACGC	GCCAAAGCGGTTCACACAC
mouse	bmp2	CCAAGAGACATGTGAGGATT	TTAGTGGAGTTCAGGTGGTC
mouse	bmp8b	CCGGGACTCCTATGGCTACT	CATCCGTCATGGCACGGTA
mouse	lsd1	ATGGATGTCACACTTCTGGA	CAAGACCTGTTACAACCATG
mouse	raptor	CTCATCGTCAAGTCCTTCAAACA	CAAGGGCATCTGGGCAAGT

Table S3. Primer sequences for shRNA

species	name	sequnence
human	lsd1sh1	CCGGGCTGAAGGCTTGGACATTAAACTCGAGTTTAATGTCCAAGCCTTCAGCTTTTTG
human	lsd1sh2	CCGGGCCTAGACATTAAACTGAATACTCGAGTATTCAGTTTAATGTCTAGGCTTTTTG
human	lsd1sh3	CCGGGCTACATCTTACCTTAGTCATCTCGAGATGACTAAGGTAAGATGTAGCTTTTTG
human	lsd1sh4	CCGGCCACGAGTCAAACCTTTATTTCTCGAGAAATAAAGGTTTGACTCGTGGTTTTTG
